# Glatiramer acetate reduces the risk for experimental cerebral malaria: a pilot study

**DOI:** 10.1186/1475-2875-8-36

**Published:** 2009-02-27

**Authors:** Peter Lackner, Andrea Part, Christoph Burger, Anelia Dietmann, Gregor Broessner, Raimund Helbok, Markus Reindl, Erich Schmutzhard, Ronny Beer

**Affiliations:** 1 Department of Neurology, Innsbruck Medical University, Innsbruck, Austria

## Abstract

**Background:**

Cerebral malaria (CM) is associated with high mortality and morbidity caused by a high rate of transient or persistent neurological sequelae. Studies on immunomodulatory and neuroprotective drugs as ancillary treatment in murine CM indicate promising potential. The current study was conducted to evaluate the efficacy of glatiramer acetate (GA), an immunomodulatory drug approved for the treatment of relapsing remitting multiple sclerosis, in preventing the death of C57Bl/6J mice infected with *Plasmodium berghei *ANKA.

**Methods and Results:**

GA treatment led to a statistically significant lower risk for developing CM (57.7% versus 84.6%) in treated animals. The drug had no effect on the course of parasitaemia. The mechanism of action seems to be an immunomodulatory effect since lower IFN-gamma levels were observed in treated animals in the early course of the disease (day 4 post-infection) which also led to a lower number of brain sequestered leukocytes in treated animals. No direct neuro-protective effect such as an inhibition of apoptosis or reduction of micro-bleedings in the brain was found.

**Conclusion:**

These findings support the important role of the host immune response in the pathophysiology of murine CM and might lead to the development of new adjunctive treatment strategies.

## Background

A major cause of morbidity and mortality of *Plasmodium falciparum *malaria is cerebral malaria (CM). It presents as a diffuse encephalopathy with alteration of consciousness, ranging from drowsiness to deep coma and is frequently accompanied by seizures [1]. Mortality is high and neurological sequelae are observed in approximately 10% of the survivors [2]. The pathophysiological mechanisms of CM are yet not fully understood. Most researchers agree that the immune response of the host is a critical factor in the pathogenesis of CM. Different aspects have been studied and in particular pro-inflammatory cytokines and activated T-lymphocytes have been shown to be related to the development of CM [3-5]. Importantly, recent studies suggest a critical role of interferon-responsive mechanisms in murine and human CM [6,7]. Therefore, immunomodulatory drugs have been considered as potential adjunctive treatment regimens for severe malaria. Despite beneficial effects in the mouse model [8,9], corticosteroids have been shown to be ineffective or even deleterious in human CM [10,11]. Similar findings were observed with TNF-alpha antagonists. While TNF-alpha blockage reduces the rate of CM in mice, the data from human trials are far from clear [12-14]. In rodents positive effects were achieved with thalidomide, a potent inhibitor of TNF-alpha and inductor of a Th2-based immune response [15,16]. Recent studies implicate, that the potent immunosuppressive agent cyclosporin A may inhibit parasite development [17] and decrease neurological complications in *Plasmodium berghei *infected mice, when applied in low dose [18]. The neuropathological alterations in murine CM resemble in some aspects the neuro-inflammatory response seen in experimental allergic encephalitis (EAE), an animal model for multiple sclerosis [19,20]. Therefore the current study was designed to evaluate the efficacy of glatiramer acetate (GA), an immuno-modulatory and potentially neuro-protective substance which is in clinical use for the treatment of relapsing remitting multiple sclerosis, for the prevention of mortality from CM.

GA, also known as copolymer 1, is a heterogeneous mix of polypeptides containing the four amino acids alanine, lysine, glutamic acid and tyrosine in definite ratios but with no uniform sequence with an average molecular weight of 5,000–9,000 daltons [21]. GA was synthesized to resemble the structure of myelin basic protein (MBP), one of the major components involved in EAE and multiple sclerosis but instead of inducing the disease, it was found to be protective [22]. GA was approved by the FDA for the treatment of relapsing remitting multiple sclerosis in 1996. GA has been shown to be of benefit in different experimental diseases models as amyotrophic lateral sclerosis (ALS) [23], Alzheimer disease [24] and experimental colitis [25]. In addition, GA exerts beneficial effects on neuronal degeneration after facial nerve axonotomy [23], graft rejection and graft versus host disease [26,27]. Recently murine HIV-1 encephalitis [28] was successfully ameliorated by GA treatment. These promising results warrant the evaluation of this drug in experimental CM.

## Methods

### Animals and treatment

A total of 72 six to eight weeks old C57BL/6J mice (Charles River, Sulzfeld, Germany) were used for this study during four subsequent experimental infections. 68 animals were infected intraperitoneally with 1 × 10^6 ^parasitized red blood cells of a homologue donor, which had been infected with frozen polyclonal stocks of *P. berghei ANKA*. Four animals were used as non-infected control animals. The clinical severity of the disease was assessed by the SHIRPA-score primary screen on baseline, day 5 post-infection and before death [29]. The primary screen comprises a battery of 40 simple tests for evaluating neuromuscular, spinocerebellar, sensory, neuropsychiatric and autonomic functions in mice by observational assessment. The scoring starts with the evaluation of undisturbed behaviour in the viewing jar. Subsequently, motor behaviour is observed and a sequence of manipulations using tail suspension is performed and visual acuity, grip strength, body tone and reflexes are recorded. Finally, autonomous functions like skin color and heart rate are assessed followed by measurement of core body temperature. The values of the respective tests were then summed up and the cumulative SHIRPA-score was calculated as described previously [29]. Healthy mice show a value of about 30 while moribund CM animals show values of about 10.

From the day of infection, animals of the treatment group (n = 34) were injected subcutaneously daily with 100 μl Copaxone (20 mg/ml GA, Sanofi-Aventis, Frankfurt, Germany) after anesthesia with Forane (Abbott Laboratories, Queensborough, UK) for two minutes on alternating injection sites. The control group (n = 34) was injected the same amount of saline (Fresenius Kabi, Graz, Austria). The course of infection was closely monitored. Core body temperature and weight were assessed daily. Parasitaemia was monitored daily by thin blood smear from tail blood. At day 4 post-infection 16 infected animals were killed to study the early course of the disease. Between day 6 and day 9 post-infection 37 of the infected mice developed signs of CM and were killed for ethical reasons as soon as their body temperature dropped to 30°C, a valid marker for imminent death [29]. Mice which did not develop cerebral malaria were killed on day 11 post-infection to minimize suffering, as it is well established that animals which do not develop CM until day 10 post-infection will die from overwhelming parasitaemia about three weeks after infection. All animals were given a lethal dose of 0.5 ml (25 mg/ml) thiopental (Biochemie, Kundl, Austria) intraperitoneally. Deeply anesthetized mice were transcardially perfused with ice-cold phosphate buffered saline (PBS) for two minutes followed by ice-cold fixative solution for 15 min with a pressure controlled syringe pump (Fresenius-Kabi, Germany). Animal studies conformed to the Austrian guidelines for the care and use of laboratory animals and were approved by the Austrian Government.

### Western blot

Animals (total n = 38, treatment = 19, control = 19) were perfused with PBS for two minutes. Samples of the forebrain and brain stem with cerebellum were processed separately as described previously [30]. The microdissected tissue was homogenized in ice-cold buffer (pH 7.5) containing 50 mM Tris-Cl, 5 mM EDTA, 50 mM NaCl, 5 mM DTT, 0.1% Np-40, 50 mM NaF, 1 mM PMSF, 1 mM Na_3_VO_4 _plus a protease inhibitor cocktail (Roche, Mannheim, Germany) and centrifuged at 18,500 g for 20 min at 4°C. Protein balanced samples were analysed using standard techniques. The bottom parts of the blots were probed with a monoclonal antibody directed against cleaved caspase-3 (Cell Signaling, Danvers, MA, USA; 1:1,000 in blocking solution) overnight at 4°C. To control and correct for equal loading, the top part of each blot was probed for alpha-tubulin (Sigma, 1:20,000 in blocking solution) overnight at 4°C. Antibody binding was visualized using enhanced chemoluminescence reagents (Lumiglo™; Cell Signaling).

### Immunohistochemistry

After perfusion with 4% PFA in PBS, brains (total n = 30, treatment = 15, control = 15) were postfixed for six hours and cryoprotected with 30% sucrose in PBS. Frozen tissues were cut into 20 μm and 40 μm thick coronal sections on a freezing cryotome (Leica Microsystems, Nussloch, Germany). 20 μm thick sections were mounted on Superfrost Plus slides (Microm International, Walldorf, Germany) and air-dried. 40 μm thick sections were kept in assorter buffer and stored at 4°C. The directly mounted slides were stained with haematoxylin-eosin (H&E) according to standard protocols and used for analysis of haemorrhagic brain area. 40 μm free-floating sections were blocked for endogenous peroxidase activity (20% methanol containing 1% H_2_O_2_) for 30 min and non-specific binding in blocking solution (20% normal goat serum, 20% bovine serum albumin in Tris-buffered saline containing 0.1% Triton X-100) for 1 hour at room temperature. All antibodies were diluted in blocking solution. Rabbit monoclonal antibody against activated caspase-3 (Cell Signaling) was diluted 1:1,000 and permitted to bind overnight at 4°C. Biotinylated goat anti-rabbit antibody (Vector Laboratories, Burlingame, CA, USA) was then applied at a dilution of 1:200 for 1 h at room temperature. Antibody binding was visualized using Vectastain ABC kit (Vector Laboratories) and diaminobenzidine as chromogen according to the manufacturer brochure and counterstained with haematoxylin. Sections without primary antibody or with primary antibody preincubated with caspase-3 blocking peptide (Cell Signaling) were equally processed to control for unspecific binding.

### Morphometric analysis

For morphometric analysis anatomical regions of interest (ROI) at +2, -6 ± 350 μm bregma were determined as previously described [29]. Stereology was applied using a computer-assisted image analysis system: Nikon E-800 microscope with a motorized stage and Stereo Investigator Software (MicroBrightField, Magdeburg, Germany). The area fraction fractionator method which is based on Cavalieri's principle was used to determine the section area and the area of haemorrhage within [31]. One section per ROI from each animal was examined systematically in a blinded way: the contour of the tissue was traced with objective ×2, analysis was performed with objective ×40 with a counting frame of 500 × 300 μm and a sampling grid area of 1,000 × 600 μm. The optical fractionator stereological method was used to count brain sequestered leukocytes and parenchymal cells immunopositive for activated caspase-3 [31]. One section per ROI from each animal was examined in a blinded way with objective ×100 with a counting frame of 70 × 50 μm and a sampling grid area of 700 × 500 μm. As brain parenchymal cells, only extravascular cells with a clear staining and morphological characteristics of neurons or glia were counted.

### Cytokine determination

Mouse serum was harvested immediately before perfusion by puncture of the right cardiac ventricle. Five different cytokines (TNF-alpha, IFN-gamma, IL-2, IL-4, IL-5) were measured simultaneously by a commercially available cytometric bead array according to the manufacturers protocol by FACS (BD Bioscience, San Jose, CA, USA).

### Statistical analysis

To test for differences in survival Kaplan-Meier curves were drawn and the treatment groups were compared using Log-rank test. Parasitaemia levels and the cumulative SHIRPA score were compared between treatment groups by repeated measures ANOVA. The relative haemorrhagic brain area, brain sequestered leukocytes and caspase-3 immunopositive brain parenchymal cells were compared between treatment groups by Wilcoxon rank sum test. Cytokine levels were compared by two-way ANOVA with time point of sampling and treatment as factors. P-values were Bonferroni-corrected for multiple comparisons. The values of IFN-gamma were logarithmically transformed to get equal variances. Calculations were done using Insightful S-Plus 6.2 (Insightful Corporation, Seattle, WA, USA), graphs were drawn by GraphPad Prism version 5.00 (GraphPad Software, San Diego, CA, USA).

## Results

### GA treated animals have a lower risk for developing CM

**Figure 1:**
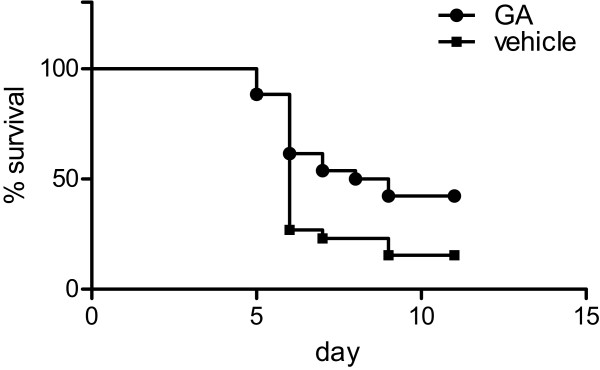
**Survival curves**. Kaplan-Meier curves for GA (circles) and vehicle (boxes) treated animals. Log-rank test yielded a statistically significant difference in the survival curves (p < 0.05).

Of the 68 infected animals 16 were killed on day 4 post-infection in order to study alterations in the early course of the disease. The remaining 52 animals entered the survival analysis. 37 of these mice developed signs of CM and were killed between day 5 and 9 post-infection when showing clinical signs of inevitable death (day 5, n = 6; day 6, n = 23; day 7, n = 3; day 8, n = 1; day 9, n = 4). The other 15 animals survived this critical period and were killed on day 11 post-infection. 15 animals (57.7%) in the treatment group developed CM compared to 22 animals (84.6%) in the control group. Kaplan Meier curves are shown in Figure 1. Log-rank test yielded a statistically significant lower risk for developing CM in the treatment group compared to the control group (p < 0.05).

### GA treatment does not affect the clinical course of the disease or parasitaemia levels

**Figure 2:**
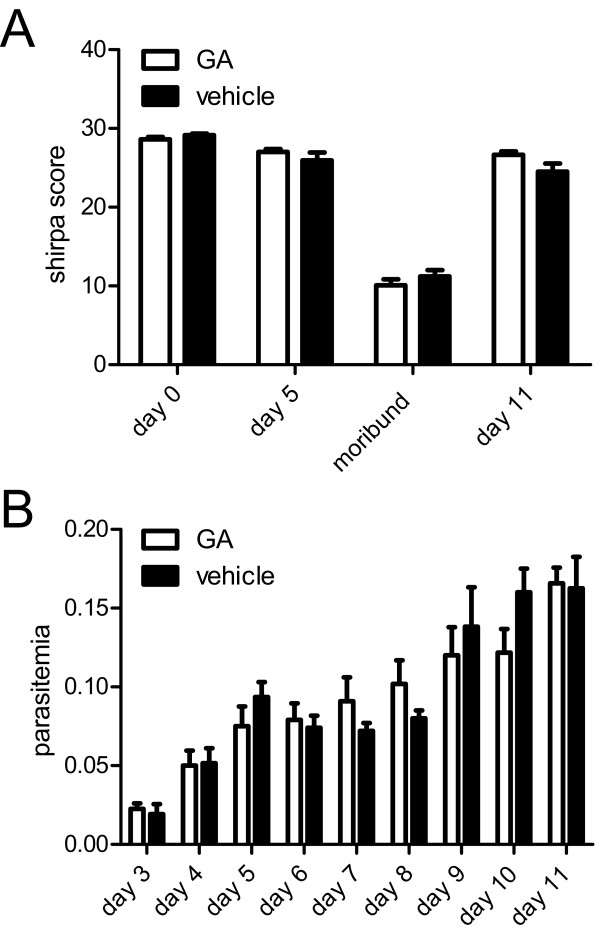
**Clinical course of the disease and parasitaemia levels**. A: Cumulative SHIRPA score of GA (open bars) and vehicle (filled bars) treated animals on day 0, 5, 11 post-infection and in moribund animals with CM. B: Course of parasitaemia of GA (open bars) and vehicle (filled bars) treated animals. No significant differences were found in the respective values of the SHIRPA score and parasitaemia between GA and vehicle treated animals. Mean values and SEM are shown.

In order to assess putative differences in the clinical course of the disease, the SHIRPA score was performed on baseline, day 5, in moribund CM animals and on day 11 post-infection in animals which did not develop CM (NCM group). No significant differences were found in the respective values of the SHIRPA score between GA and vehicle treated animals (Figure 2A). There was a trend towards a higher/better SHIRPA score in GA treated NCM animals on day 11 post-infection (p = 0.056; Figure 2A). Parasitaemia levels were also compared and did not yield significant differences (Figure 2B).

### GA treatment does not result in reduction of microhaemorrhages or brain parenchymal apoptosis

In order to reveal putative effects of GA treatment on the neuropathology of CM, conventional H&E staining, immunohistochemistry and western blot analysis for activated caspase-3 was performed. Qualitative inspection of H&E stained sections did not reveal differences between GA and vehicle treated animals. All moribund CM animals showed the typical neuropathology of CM (i.e. haemorrhage, sequestration, perivascular oedema). In the early course of the disease (on day 4 post-infection) and in infected animals without CM (NCM animals, 11 days post-infection) no signs of microhaemorrhages were observed.

**Figure 3:**
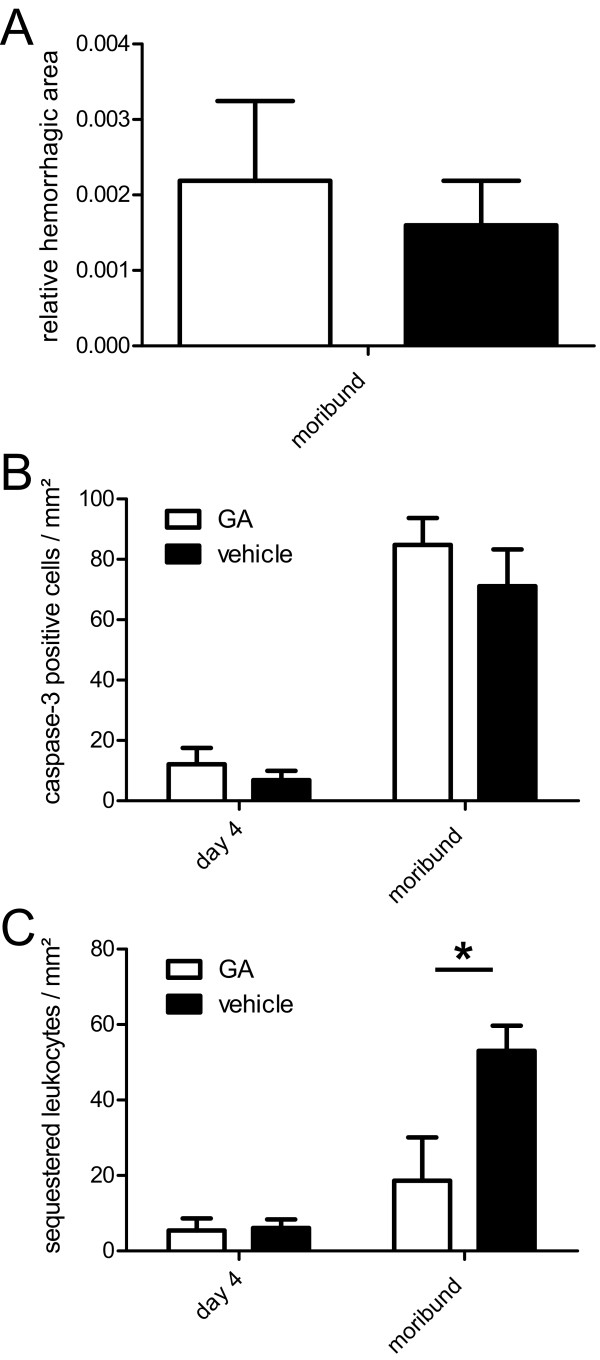
**Histological analysis of microhaemorrhages, activated caspase-3 positive cells and brain sequestered leukocytes**. Stereological analysis of histology of GA (open bars) and vehicle (filled bars) treated animals. A: Relative of microhaemorrhages affected brain area in moribund CM animals. B: Total number of parenchymal cells immunopositive for activated caspase-3 on day 4 post-infection and in moribund CM animals. C: Total number of brain sequestered leukocytes on day 4 post-infection and in moribund CM animals. Mean values and SEM are shown.

Histopathological changes were quantified by stereological methods. In moribund CM animals the relative haemorrhagic brain area was not significantly different between GA and vehicle treated animals (Figure 3A). The relative number of activated caspase-3 immunopositive brain parenchymal cells on day 4 post-infection and in moribund CM animals was not significantly different between GA and vehicle-treated animals (Figure 3B). On day 11 post-infection no activated caspase-3 immunopositive cells could be observed. Densitometric analysis of Western blots did not show significant difference for activated caspase-3 between GA and vehicle treated mice on day 4 post-infection or in moribund animals.

The relative number of brain sequestered leukocytes on day 4 post-infection was not significantly different between GA and vehicle-treated animals (Figure 3C). In moribund animals GA treated animals showed a significantly lower number of brain-sequestered leukocytes.

### GA treated animals show lower IFN-gamma levels in the early disease course

**Figure 4:**
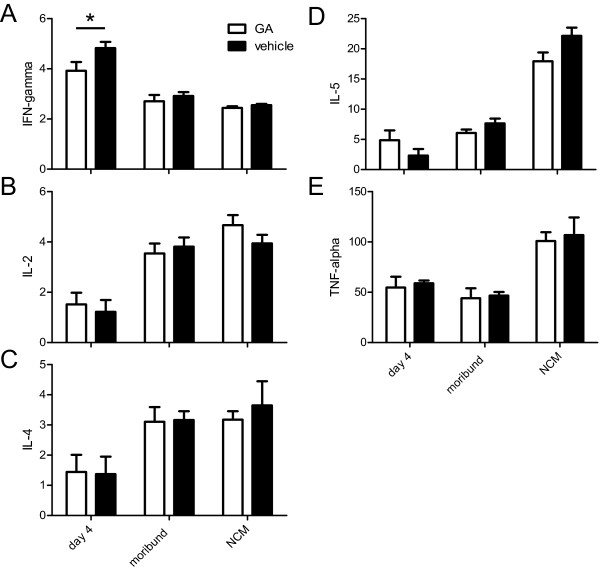
**Cytokine levels in sera**. Cytokine levels in sera at day 4, 11 post-infection and in moribund animals with CM (pg/ml). (A: Interferon-gamma, B: Interleukin-2, C: Interleukin-4, D: Interleukin-5, E: Tumor-necrosis factor-alpha). On day 4 post-infection GA treated animals showed a significantly lower level of IFN-gamma than vehicle treated animals (A; *, p < 0.05). IFN-gamma levels on day 4 were significantly higher than the levels in moribund animals or in animals on day 11 post-infection (A; p < 0.001). TNF-alpha levels on day 4 and in moribund animals were significantly lower than on day 11 post-infection (E; p < 0.001). IL-2, IL-4, IL-5 levels on day 4 were significantly lower than the levels in moribund animals or in animals on day 11 post-infection (B-D; p < 0.001). IL-5 levels in moribund animals were significantly lower than in animals on day 11 post-infection (D; p < 0.001). Mean values and SEM are shown.

The immuno-phenotype of animals was determined on day 4, in moribund CM animals, and on day 11 post infection in NCM animals by measuring 5 different cytokines in sera. On day 4 post-infection GA treated animals showed a significantly lower level of IFN-gamma (p < 0.05, Figure 4A). There were no significant differences in the levels of IL-2, IL-4, IL-5 or TNF-alpha (Figure 4B, C, D, E) between the treatment groups. IFN-gamma levels on day 4 were significantly higher than the levels in moribund animals or in animals on day 11 post-infection (Figure 4A, p < 0.001, Bonferroni corrected). TNF-alpha levels on day 4 and in moribund animals were significantly lower than on day 11 post-infection (Figure 4E, p < 0.001, Bonferroni corrected). IL-2, IL-4, IL-5 levels on day 4 were significantly lower than the levels in moribund animals or in animals on day 11 post-infection (Figure 4B, C, D, p < 0.001, Bonferroni corrected). IL-5 levels in moribund animals were significantly lower than in animals on day 11 post infection (Figure 4D, p < 0.001, Bonferroni corrected).

## Discussion

The current study was conducted to evaluate the efficacy of the immuno-modulatory agent GA in preventing the death of C57Bl/6J mice infected with *P. berghei *ANKA due to CM. A statistically significant lower risk for developing CM in GA treated animals could be demonstrated. The drug had no effect on the course of parasitaemia. The mechanism of action seems to be an immuno-modulatory effect since lower IFN-gamma levels were observed in GA-treated animals in the early course of the disease which also led to a lower number of brain sequestered leukocytes in treated animals. No direct neuro-protective effect such as inhibition of caspase-3 mediated apoptosis or reduction of microbleedings in the brain was observed.

GA is able to bind to MHC class 2 molecules on antigen presenting cells and competes with myelin basic proteins (MBP) and other myelin associated antigens such as proteolipid protein (PLP) and myelin-oligodendrocyte glycoprotein (MOG) for this binding or even displaces proteins from the binding site [32]. This mechanism reduces the ability of MBP/MHC complexes to bind to and activate myelin reactive T-cells [33]. Importantly, dendritic cells have recently been shown to be tightly involved in the induction of murine CM [34].

Sequestered leukocytes are recognized to be causally related to the pathogenesis of murine CM. The onset of neurological signs and symptoms is paralleled by the sequestration of CD8+ T-lymphocytes in the brain, and depletion of these cells confers protection against CM. Accumulation of activated T cells in the brains of CM mice has been shown by different experimental approaches [5,35]. Beside T-lymphocytes, monocytes are the major cell population sequestering on the murine cerebral endothelium in fatal CM [36]. Exposure of monocytes to GA inhibits their activation by pro-inflammatory cytokines (in particular by IFN-gamma) in vivo and in vitro [37]. Further, IFN-gamma release is suppressed by GA treatment [38,39]. IFN-gamma has recently been identified to be a critical cytokine in the pathogenesis of murine CM [6,40]. Hence, the decreased IFN-gamma levels on day 4 post-infection in treated animals in addition to a relative IFN-gamma resistance of brain sequestered monocytes could be the mechanism of action conferring protection from CM in our mouse model. Indeed, in the current study, a lower number of brain sequestered leukocytes was observed in CM animals treated with GA. Whereas no subtype characterization of brain sequestered leukocytes was achieved in the study presented, this important issue is currently under investigation in our laboratory to further elaborate the exact mechanisms of GA mediated protection from murine CM. Nevertheless, the data presented here, besides uncovering a potential adjunctive treatment, allow new insights into the immuno-pathogenesis of experimental CM.

The neuro-protective effects of GA have been demonstrated in several animal studies. In the EAE model and in an optic-nerve injury model GA protected neurons from demyelination, axonal damage and degeneration [41,42]. Further, anti-apoptotic effects have been reported for GA [43]. In a rat model for elevated intraocular pressure, apoptosis of retinal ganglion cells was reduced by GA. In the current study apoptosis was investigated by immunostaining of activated caspase-3 in brain sections and brain homogenates. No significant differences were observed between the two treatment groups. Therefore, the protective effect of GA is most likely not mediated by inhibition of caspase-3 induced apoptosis. Noteworthy, in a recent study administration of EPO protected mice from death of CM and the effect of EPO was also not related to the inhibition of apoptosis [44]. However also conflicting data on the effect of EPO on neuronal apoptosis is existing [45]. In previous studies we demonstrated that neuronal apoptosis and the occurrence of parenchymal microhaemorrhages are mainly features of late murine cerebral malaria [29,30]. In the present study, mice which developed clinical signs of CM invariably died regardless of their treatment status, showing the typical histopathological hallmarks of murine CM. This is a further argument that the effect of GA was rather immuno-modulation during the early course of the disease and not neuro-protection aiming at later stages of CM.

It should be noted that GA did neither change the course of parasitaemia nor the clinical course of the disease. This is of importance since a new adjunctive drug for treating severe complicated malaria must not interfere with parasite clearance through schizonticidal medication [46]. Further, clinical presentation was not affected in the early course of the disease which can be regarded as an indicator that GA administration is obviously well tolerated in mice infected with *P. berghei *ANKA. In general, subcutaneous treatment with GA seldom causes severe side effects [23]. The most frequent adverse events are local reactions on the injection site [47]. No higher incidence of infections is reported, an issue which is critical with respect to putative future studies in humans in view of concomitant infections in malaria endemic areas [48].

## Conclusion

In conclusion, the present pilot study provides direct evidence that GA, a drug which has been safely administered to patients with relapsing remitting multiple sclerosis for several years, reduces the risk for experimental cerebral malaria. The mechanism of action seems not to be neuro-protection through inhibition of caspase-3 dependent apoptosis or inhibition of parasite growth but rather immuno-modulation in the early course of the disease. Further studies are currently performed to evaluate the exact mechanisms of GA mediated immuno-modulation in CM and to determine the most effective dosing and timing of treatment. The current findings support the important role of the host immune response in the pathophysiology of murine CM and might lead to the development of new adjunctive treatment strategies.

## Competing interests

The authors declare that they have no competing interests.

## Authors' contributions

PL, AP, CB, AD performed all experiments. PL, GB, RH, MR, RB analyzed the data. PL, ES, RB had the idea and designed the study. All authors participated in the interpretation, and writing of the paper and read and approved the final manuscript.
